# Pathobiology of Respiratory Syncytial Virus (RSV)

**DOI:** 10.3390/vaccines8030367

**Published:** 2020-07-09

**Authors:** Ralph A. Tripp, Paul S. McNamara

**Affiliations:** 1 Department of Infectious Diseases, University of Georgia, Athens, GA 30602, USA; 2 Department of Child Health, University of Liverpool, Liverpool L69 3BX, UK; mcnamp@liverpool.ac.uk

Respiratory Syncytial Virus (RSV) is the leading cause of lower respiratory tract illness in infants and affects the elderly and the immune-compromised. RSV infection can cause pulmonary obstruction and disease, but the mechanisms contributing to these outcomes remain elusive. Despite the major health burden caused by infection, there is currently no licensed RSV vaccine and treatments are limited. The Special Issue on “Pathobiology of Respiratory Syncytial Virus (RSV)” covers the attributes of disease pathogenesis, immunity, and vaccine development. In an article by Pedro Piedra, “Antibody Response to the Furin Cleavable Twenty-Seven Amino Acid Peptide (p27) of the Fusion Protein in Respiratory Syncytial Virus (RSV) Infected Adult Hematopoietic Cell Transplant (HCT) Recipients” [1], the antibody response in HCT recipients to an immunodominant peptide made by furin cleavage of the F protein, p27, was shown to be detectable in both serum and nasal wash samples; however, 30% of RSV-infected HCT recipients had a substantial decrease in the mucosal anti-p27 antibody. These findings influence current vaccine development efforts. The type of RSV vaccine and its safety and immunity are considerations in vaccine development. In an article by Trudy Morrison, “Comparisons of Antibody Populations in Different Pre-Fusion F VLP-Immunized Cotton Rat Dams and Their Offspring”, [2] the efficacy of virus-like particle (VLP) vaccine candidates for RSV containing a stabilized pre-fusion F protein or mutation-stabilized pre-fusion proteins was examined in VLP-immunized cotton rats and their offspring. The study showed that the immunization of pregnant animals with one of these pre-fusion F VLPs increased the serum-neutralizing antibody responses and protection from RSV challenge. The results suggest the importance of a stabilized pre-fusion F in formulating a maternal vaccine for protection against RSV in neonates. In a review article by Fernando Polack, “A Durable Relationship: Respiratory Syncytial Virus Bronchiolitis and Asthma past Their Golden Anniversary” [3], he summarizes the literature exploring the association between RSV lower respiratory tract infection in infancy and subsequent recurrent wheezing and pediatric asthma. The review discusses the evidence that RSV bronchiolitis is an assortment of entities presenting clinically as a syndrome with partial differences in phenotypes and defined differences in disease mechanisms. Understanding these pathways to acute disease is important in order to understand the beginning and evolution of chronic wheezing disorders. In another review article on “Function and Modulation of Type I Interferons during Respiratory Syncytial Virus Infection”, [4] Stephen Varga discusses the implications for RSV vaccine design and the role of Type I IFN and the innate immune response, as well as using Type I IFNs in therapeutic and preventative strategies for RSV. Additionally, the importance of dendritic cells and their production of type I IFNs is considered with increased RSV disease severity. Finally, the Special Issue ends with a commentary by Ralph Tripp and Ultan Power on “Original Antigenic Sin and Respiratory Syncytial Virus Vaccines” [5], which discusses how origin antigenic sin (OAS) may affect RSV vaccine development by modifying the memory response to the first RSV exposure or vaccine antigen. The contention of the article is that the propensity of the immune response to favor responses to an original antigen over a related or new antigen affects the response to subsequent RSV infections. Notably, the article coalesces many of the features covered by the other articles in the Special Issue, particularly on vaccine development. This Special Issue reveals many of the major challenges in the pathobiology of RSV and discusses some of the challenges in RSV vaccine development, despite decades of ongoing efforts to develop a safe and efficacious RSV vaccine.
